# Use of a participatory quality assessment and improvement tool for maternal and neonatal hospital care. Part 1: Review of implementation features and observed quality gaps in 25 countries

**DOI:** 10.7189/jogh.10.020432

**Published:** 2020-12

**Authors:** Giorgio Tamburlini, Alberta Bacci, Marina Daniele, Stelian Hodorogea, Dalia Jeckaite, Gelmius Siupsinskas, Emanuelle Pessa Valente, Paola Stillo, Francesca Vezzini, Maurice Bucagu, Ornella Lincetto

**Affiliations:** 1 Centro per la Salute del Bambino, Trieste, Italy; 2 International perinatal care consultant, Trieste, Italy; 3 Department of Women and Children's Health, Faculty of Life Sciences and Medicine, King's College London, UK; 4 Department of Obstetrics and Gynecology, State University of Medicine and Pharmacy, Chisinau, Moldova; 5 International midwifery and perinatal care consultant, Panevezys, Lithuania; 6 International perinatal consultant, Geneva, Switzerland; 7 Institute for Maternal and Child Health IRCCS Burlo Garofolo, WHO Collaborating Centre for Maternal and Child Health, Trieste, Italy and Instituto de Medicina Integral Fernando Figueira, Recife, Brazil; 8 Paediatric Emergency Department and Trauma center Meyer Hospital, Florence, Italy; 9 Instituto de Medicina Integral Fernando Figueira, Recife, Brazil; 10 WHO Department of Maternal, Newborn, Child, Adolescent Health and Ageing, Geneva, Switzerland; 11 WHO Department of Maternal, Newborn, Child, Adolescent Health and Ageing, Geneva, Switzerland

## Abstract

**Background:**

A substantial proportion of maternal and neonatal mortality and morbidity is attributable to gaps in quality of care. A systematic, standard-based tool for quality assessment and improvement for maternal and neonatal hospital care (QA/QI MN tool) was developed in 2009 by the World Health Organization (WHO). The tool guides the assessment process along the whole continuum from admission to discharge, collects the views of the recipients of care and engages hospital mangers and staff in identifying gaps and drafting an action plan.

**Methods:**

Publications describing use of the WHO QA/QI MN tool from 2009 to 2017 and reports retrievable from WHO or other development partners’ websites were searched and considered for inclusion in the review. Only assessments of hospitals were considered. Quality gaps were classified as regarding case management in maternal care, case management in neonatal care, hospital infrastructure, hospital policies and according to severity and frequency. Quotations from women regarding key issues in effective communication, respect and dignity, emotional support and costs incurred were selected.

**Results:**

In the period 2009-2017, use of the WHO QA/QI MN tool was documented in 25 countries, belonging to Central and Eastern Europe (8), Central Asia (4), Sub-Saharan Africa (11), Latin America (1) and Middle East (1). Overall, 133 hospitals were assessed. The tool allowed to identify in great detail serious quality gaps including: insufficient or incomplete adherence to recommended evidence-based procedures for normal childbirth and maternal and neonatal complications; excess of inappropriate or unnecessary interventions; insufficient infection control; failure to provide respectful care, adequate communication and emotional support to mothers and babies; poor use of information generated locally to analyse processes and outcomes. These gaps were observed in all countries. Significant differences were observed among facilities belonging to the same health systems, ie, with very similar staffing, infrastructure and equipment.

**Conclusions:**

The experience made, the largest of this kind, provides comprehensive and detailed insight into the existing quality gaps in a wide variety of settings. QI cycles at facility level should be primarily based on assessments made by multidisciplinary teams of professionals to identify the parts of the care pathways which require improvement through a participatory approach involving managers, staff and patients.

A substantial proportion of maternal and neonatal mortality and morbidity is attributable to gaps in quality of care (QoC) [1,2]. Under WHO leadership, the Global Strategy for Women's, Children's and Adolescent's Health 2016-2030, the Every Newborn Action Plan and the Strategy for Ending Preventable Maternal Mortality were developed and recognize that investments in QoC are an imperative in order to translate access into effective coverage of essential interventions [3-5]. To intensify efforts in improving QoC, WHO developed a strategic framework [6], accompanied by standards for maternal and neonatal health care (MNHC) [7]. A network to improve quality, equity and dignity of MNHC was launched [8]. However, progress in identifying and implementing at scale effective approaches to improve the quality of MNHC is still too slow. A variety of tools and methods have been developed and used but to a much lesser extent evaluated for feasibility and results [9-11]. Challenges include: the scalability of tools; their ability to assess quality along the whole continuum of care with sufficient granularity to allow precise identification of problems, their causes and solutions; the use of data to inform and start quality cycles; and the capacity of engaging health professionals and managers in a participatory process from assessment to action, so to build a QoC culture among professionals and managers [12].

A systematic, action-oriented and participatory tool for quality assessment and improvement for MN hospital care (QA/QI MN tool) was developed in 2009 as part of the European Strategy for Making Pregnancy Safer [13,14], updated in 2014 [15] and adapted by WHO Headquarters for global use with an added section on pediatric care [16]. The tool has been used in a wide variety of countries and health system contexts, mainly within country-wide initiatives upon request by Ministries of Health (MoHs) and supported by UN Agencies (WHO, UNICEF, UNFPA) and other development partners, and in a few cases as single-facility exercises in hospitals supported by NGOs. The extent of the experience made with the tool offers the opportunity for a review of key implementation features, observed quality gaps over the continuum of perinatal care, improvements that were observed at reassessment and key factors influencing change. This first paper describes where, when and how the tool was implemented and describes the MNHC quality gaps that emerged from the assessments.

## METHODS

### Assessment tool

The WHO QA/QI MN tool includes five sections. Four steer the evaluation on: hospital support services; case management; hospital policies and organization of services; experience of care by staff members, pregnant women and mothers. A fifth one suggests suitable templates for presenting the findings, facilitating feedback to regional/national level, and drafting an action plan for quality improvement. The tool is based on standards and guidelines developed by WHO. Adherence to standards is assessed through several hundred items, most of them addressing case management of normal birth as well as maternal and neonatal complications.

A detailed description of the tool’s structure and assessment methods can be found in Appendix S1 of the **Online Supplementary Document**.

### Literature search

We searched for publications and reports that described the use of the WHO QA/QI MN tool since 2009. All papers published in peer reviewed journals and reports retrievable from WHO or other development partners’ websites were included. Unpublished reports were also searched through collaboration with WHO headquarters, WHO Regional offices and international experts involved in the assessments.

Reports and publications were considered for inclusion in the review if they contained: a) an analytical description of methods, including tool adaptation, composition of the international and national assessors’ team and characteristics of the facilities being assessed; b) detailed information on the main features of the assessment process, including the involvement of staff and mothers, and of its results; c) a summary description of recommended actions. Only assessments of district or regional level hospitals were considered. Health Centers or equivalent facilities, which were assessed together with hospitals in a few countries, were not included. Overall, 2 published papers, 1 submitted paper and 27 reports were identified. 2 out of the latter were not included as not satisfying the second selection requisite. Decisions about whether identified publications met the requirements for being included in the review were based on scrutiny independently made by two of the Authors (AB, GT). Information retrieved from publications and reports included: country, number of hospitals, year of the assessment, composition of the assessment team, leading organizations, duration of the assessment, reported quality gaps.

### Identification, framing and reporting of quality gaps

In order to produce a functional synthesis of the huge amount of information regarding the quality gaps emerging from the assessments, we developed a framework that consists of four sections: 1) issues regarding case management in maternal care, 2) issues regarding case management in neonatal care, 3) issues regarding hospital infrastructure and 4) issues regarding hospital policies. A distinction between infrastructure and policies was made because whereas several policy-related issues can be addressed at facility level (eg, production of clinical protocols, data collection and use, infection prevention procedures, etc.), only few issues related to infrastructure and staffing depend on decisions at facility level.

For each of these four sections quality gaps were identified with reference to WHO maternal and neonatal care standards [7] and categorized according to two criteria: severity and frequency. A gap in a specific area of care was considered “severe” if, according to the tool scoring system, the assessment indicated “*inadequate care” or “very poor care”,* and “frequent” when observed in at least 1/3 of all the assessed facilities, irrespective of the country. While fully aware that each country context is different, we decided to identify the most common and pressing concerns across countries and provide a semiquantitative overview of the findings. Quantitative statistics based either on countries or on hospitals as denominators were not used because the great variability of the number of hospitals in each country (ranging from 1 to 28) would make percentages meaningless. For each identified severe and frequent gap, we chose to provide at least one illustrative example drawn from one or more reports.

While it was not feasible to conduct a full analysis of the qualitative information from interviews with service users and health workers, because transcripts were rarely available and quality of the reporting heterogeneous, we chose to present only a selection of quotations from pregnant and postpartum women that were relevant to the three areas of experience of care included in the WHO vision of quality of maternal and neonatal care, namely effective communication, respectful and dignified care and emotional support [6] to which a fourth component, related to the costs incurred by women and their families, was added.

These analyses were independently made by two authors (AB and GT) according to the above criteria and serious and frequent gaps were then identified upon mutual agreement.

### Role of the funding source

The review was funded by WHO (Department of Maternal, Neonatal, Child and Adolescent Health and Aging, Geneva). The funding source contributed to the study design, data collection, review of the draft manuscript and decisions about submitting the paper for publication.

## RESULTS

### Coverage and implementation features

In the period 2009-2017, use of the WHO QA/QI MN tool was documented in 25 countries, belonging to Central and Eastern Europe (8 countries), Central Asia (4), Sub-Saharan Africa (11), Latin America (1) and Middle East (1). Overall, 133 hospitals were assessed, with numbers of facilities assessed in each country ranging from a single facility to a representative sample to all hospitals (**Table 1**).

**Table 1:** Use of the WHO QA/QI MN tool over the period 2009-2017

Country	Year of assessment	No. of hospitals	Involved authorities and agencies
Albania	2009	3	MoH, WHO, Spanish Government Aid Agency
Armenia	2012	4	MoH, UNFPA
Benin	2017	6	MoH, WHO
Brazil	2015	6	CNP, Pernambuco Health Authorities
Burkina Faso	2016-2017	29	MoH, WHO, UNICEF
Chad	2016	3	MoH, WHO
Congo Brazzaville	2016	9	MoH, WHO, UNFPA, UNICEF
Cote d’Ivoire	2015	5	MoH, WHO
Ethiopia	2012	1	Doctors with Africa – CUAMM
Gaza strip (OPT)	2009	3	MoH (Gaza), WHO
Georgia (Abkhazia)	2017	3	UNICEF and Local Health Authorities
Kazakhstan	2009	4	MoH, WHO, European Union
Kyrgyzstan	2012	3	MoH, WHO, UNFPA, UNICEF
Kosovo	2011	4	MoH,WHO, UNFPA, UNICEF, Luxemburg Gov.t,
Malawi	2015	6	MoH, WHO, UNICEF, LSHTM
Moldova*	2013-2016	6	MoH, WHO, Swiss ADC
Montenegro	2011	5	MoH, UNICEF
Niger	2016	5	MoH, WHO
Swaziland	2015	5	MoH, WHO, UNFPA, UNICEF
Tanzania	2012	1	Doctors with Africa – CUAMM
Tajikistan	2011	4	MoH, USAID, WHO, UNFPA, GTZ
Turkmenistan	2009	3	WHO/UNFPA/Zdrav Plus-USAID
Uganda	2012	1	Doctors with Africa – CUAMM
Ukraine	2011	7	WHO, USAID/JSI/MIHP
Uzbekistan	2010	4	MoH, UNICEF, WHO, EU

ADC – Agency for Development and Cooperation, MIHP − Mother and Infant Health Project, Conselho Nacional de Pesquisa (National Research Council), OPT – Occupied Palestinian Territory, WHO – World Health Organization

*Includes Transnistria.

Over the period 2009-2018, three versions of the tool have been used: the 2009 version, the 2014 revised version and a third version adapted by WHO Headquarters. The three versions were used depending on their availability at the time of first assessment and on country, with the third version used in francophone Sub-Saharan countries and Malawi.

In 22 cases the assessment involved a sample of hospitals as part of a country-wide (or State-wide, as in the case of Brazil) initiative, with involvement of MoH or equivalent State health authorities. WHO was the lead agency or among the organizing agencies in 18 countries. In Uganda, Tanzania and Ethiopia the assessments were made in only one hospital and were led by a NGO, Doctors with Africa CUAMM, with the involvement of District and Regional health authorities. A national assessment team was supported by international experts in all cases. A feedback on main gaps based on the scoring system and indications on priority actions to be included in a draft plan of action were provided in all cases. In 4 out of 25 countries, clear evidence of inclusion of experience of care based on the interviews with pregnant women and mothers could not be found in the reports. On average, the assessment process required one to two days for the training of the national or local assessment team, and two and a half to three days for the assessment, including the feedback. Costs varied depending on the logistics of each assessment, and included expenses related to the international team, such as travel, lodging and fees, usually covered by international agencies, and those related to the national assessors, including travel, lodging and per diem for public servants and usually covered by national authorities.

### Synthesis of the observed quality gaps

The main quality gaps emerging from the assessments are summarized in **Table 2****,** **Table 3****,** **Table 4** and **Table 5****.** In accordance to the framework described in the methods section, the first two tables report the gaps related to case management, while the latter two describe the gaps related to infrastructure and policies, respectively. Reference to the relevant WHO MNHC eight quality standards [7] is indicated in the title of each table. Main areas where serious gaps were identified in at least 1/3 of the health facilities are reported in the second column and detailed examples for each area in the third column.

**Table 2:** Summary of main quality gaps emerging from the baseline assessments: provision of effective, safe and respectful care to pregnant women and mothers (compared with WHO standards 1, 2, 4, 5 and 6) [7]

WHO quality standards	Areas where serious gaps were identified in at least 1/3 of the health facilities	Examples of gaps
**Standard 1: Every woman and newborn receives routine, evidence-based care and management of complications during labour, childbirth and the early postnatal period, according to WHO guidelines**	Monitoring of maternal and foetal conditions during labour and birth	Partographs often filled in *a posteriori.*
		Fetal Heart Rate (FHR) rarely auscultated more than 4-hourly, usually at time of vaginal examinations, when missing recordings are frequently filled in.
		Maternal heart rate never taken alongside the FHR, and never recorded.
		In-out fluids and medications rarely recorded.
	Excess and/or inappropriate intervention	Excess of episiotomies.
		Potentially harmful procedures: catheterization shortly after delivery in the absence of postpartum haemorrhage, routine vaginal examination after vaginal delivery for the extraction of clots.
**Standard 2: The health information system enables use of data to ensure early, appropriate action to improve the care of every woman and newborn**		Unnecessary use of combination of drugs, eg, antihypertensive drugs.
	Early identification and management of emergencies	Women left without assessment of progress for over 5 h.
		Insufficient measurement of blood loss and inappropriate management of 3rd stage of labour.
		Use of IV oxytocin to augment labour not recorded on partograph, nor anywhere else. Oxytocin used in absence of close monitoring, including FHR.
		Vaginal delivery after previous Caesarean Section (CS) offered, but without closer monitoring of maternal and foetal conditions.
		Lack of basic emergency procedures such as correctly positioning the patient.
		No coordinated reaction when an emergency occurs.
	Management of complications	Inappropriate/outdated management of severe preeclampsia.
		Administration of Magnesium Sulphate without indication of timing, delays between prescription and administration of drugs, blood and urine tests requested but results not recorded.
		Women who experienced complications discharged too soon.
	Caesarean section indications and procedures	General anaesthesia used for CS.Indications for CS not reported, sometimes questionable: (eg, obstructed labour when the **partograph action line has not been crossed; foetal distress when FHR is not measur**ed).
**Standard 4: Communication with women and their families is effective and responds to their needs and preferences**	Effective communication	Women not told about indications for CS and not given information about their baby’s conditions.
		Women poorly informed about appropriate care after discharge.
		Women not involved in decisions regarding care for them and their baby.
**Standard 5: Women and newborns receive care with respect and preservation of their dignity**	Respect and dignity	Freedom to move in labor not ensured
		Lack of privacy during birth.
		Disrespectful attitude, inadequate consideration of feelings.
		Users’ needs neglected in ward lay-out.
**Standard 6: Every woman and her family are provided with emotional support that is sensitive to their needs and strengthens the woman’s capability**	Emotional support	Companion’s presence not allowed/ encouraged during labour and birth.
		One-to-one care not ensured.

**Table 3:** Summary of main gaps emerging from the baseline assessments: provision of effective, safe and respectful care to newborn babies (compared with WHO standards 1 and 5) [7]

WHO quality standards	Areas where serious gaps were identified in at least 1/3 of the health facilities	Examples of gaps
**Standard 1: Every woman and newborn receives routine, evidence-based care and management of complications during labour, childbirth and the early postnatal period, according to WHO guidelines**	Early mother-baby contact and immediate initiation of breastfeeding	Early skin-to-skin contact not ensured.
		Initiation of breastfeeding within the first hour not ensured.
	Resuscitation preparedness and procedures	Preparedness for newborn resuscitation (skills and equipment) insufficient.
		Apgar score not applied properly.
		Newborn resuscitation not started according to the recommended algorithm.
	Care for premature/Low Birth Weight Babies	Kangaroo care not implemented.
		Inadequate nutrition of prems/LBW/sick babies, feeding needs not calculated.
	Excess and/or inappropriate interventions	Unnecessary nasogastric aspiration..
		Unjustified use of drugs based on inappropriate diagnosis of perinatal asphyxia
	Early identification and monitoring of risk conditions and complications	Poor recording of vital signs.
		Poor recognition of signs of infection.
		Monitoring of women and baby in delivery room (first 2 h) not ensured.
	Management of complications	Delayed diagnosis of infection.
		Over-diagnosis of infection.
**Standard 5: Women and newborns receive care with respect and preservation of their dignity**	Mother-baby bonding	Unjustified separation at birth.
		Babies kept separated from mothers without medical reasons for most of the time.
	Pain prevention and relief	Excess of painful diagnostic procedures with no attention to pain prevention.
		No attention paid to guarantee a quiet silent environment
		Mothers not involved in care of sick newborn babies.

WHO – World Health Organization

**Table 4:** Summary of main quality gaps emerging from the baseline assessments: human resources and infrastructure (compared with WHO standards 7 and 8) [7]

WHO quality standards	Areas where serious gaps were identified in at least 1/3 of the health facilities	Examples of gaps
**Standard 7: For every woman and newborn, competent, motivated staff are consistently available to provide routine care and manage complications.**	Human resources number and skills mix	Insufficient number of midwives and neonatal nurses.
		Newly graduated staff utilized in neonatal intensive care unit without supervision.
***Standard 8: The health facility has an appropriate physical* environment, with adequate water, sanitation and energy supplies, medicines, supplies and equipment for routine maternal and newborn care and management of complications.**	Hygienic facilities and waste disposal	Insufficient / inadequate toilets.
		Lack of sufficient washing facilities for patients.
		Unsafe disposal of waste.
	Water and energy	Frequent power breakdown.
		Discontinuous availability of running water and warm water.
	Physical structure	Insufficient number of individual delivery rooms, delivery room layout not ensuring privacy.
		Operating theatre far from labour ward and delivery area.
		No specific dedicated area exists for the care of the sick/ill/premature baby.
	Essential medicines	Irregular procurement and stock.
	Essential equipment and supplies	Poor maintenance of equipment.
		Lack of basic equipment (wall clock, thermometer, etc.).
		Underutilization of up-to-date equipment.
		Substandard laboratory services, lack of microbiology laboratory even at referral level.
		Lack of blood bank even at tertiary level.

**Table 5:** Summary of main gaps emerging from the baseline assessments: policies (compared with WHO standards 1, 2, 3, 5 and 7) [7]

WHO quality standards	Areas where serious gaps were identified in at least 1/3 of the health facilities	Examples of gaps
**Standard 1: Every woman and newborn receives routine, evidence-based care and management of complications during labour, childbirth and the early postnatal period, according to WHO guidelines.**	National clinical guidelines and local protocols	Lack or poor access to clinical guidelines for case management.
		Lack of local protocols.
		Lack of essential drug list.
	Infection prevention and control	Inappropriate hand washing by staff.
		Sterile gloves used inappropriately as a substitute for hand washing.
		Mosquito nets available but patients not encouraged to use them.
		Inadequate registration of nosocomial infections.
		Lack of guidelines for appropriate use of antibiotics both in obstetrics and neonatology.
**Standard 2: The health information system enables use of data to ensure early, appropriate action to improve the care of every woman and newborn**	Data collection and use	Poor information system.
		Poor local use of data for care provision and organization.
		Substandard or poorly filled medical records.
	Periodical perinatal audit	Lack of medical record for newborn babies.
		Absence of maternal and perinatal case reviews.
		Insufficient capacity to use maternal and perinatal audits for identifying and addressing gaps in organization, skills or procedures.
**Standard 3: Every woman and newborn with condition(s) that cannot be dealt with effectively with the available resources is appropriately referred**	Perinatal referral system	No criteria-based functional referral system for mother and newborns.
		Insufficient communication among different levels of care.
**Standard 5: Women and newborns receive care with respect and preservation of their dignity**	Access to care	Official and unofficial fees requested.
		Need to pay for drugs and consumables (cannulas, catheters, syringes, gloves, etc).
**Standard 7: For every woman and newborn, competent, motivated staff are consistently available to provide routine care and manage complications.**	Human resources development and/or deployment	Insufficient number of midwives
		Lack of continuous professional development, especially for paramedics.
		Insufficient involvement of midwives into care provision and organization.

#### Women’s views

At least ten semi-structured interviews were conducted in each facility with pregnant and postpartum women by trained professionals who were part of the assessment teams. Information was collected on all aspects of care. Almost all assessment reports mentioned women’s views, and the majority reported direct quotations. Many women expressed positive remarks and most declared to trust the health services and the health providers. There were several comments acknowledging staff attitude and support. On the other hand, many women reported that the communication with service providers was insufficient and felt insufficiently informed about the progress of labour, the conditions of the baby and post discharge care, including family planning. They were not happy with fees and the need to buy consumables, drugs or food. **Table 6** reports a sample of quotations related to perceived quality gaps, with reference to key dimensions of experience of care [6] and incurred costs.

**Table 6:** Examples of women’s views about perceived quality gaps in their experience of care during labour, childbirth and postpartum care with respect to effective communication, respect and dignity, emotional support and incurred costs [6]

Main area [6]	Women’s quotes
Effective communication	*“I wish I had not been only checked, but given information about breastfeeding, contraceptive methods after childbirth, how to take care of my child, and more...”*
	*“I would like to come to the reception and someone to tell me about labour, what will happen to me, anaesthetic technique, how to behave”*
Respect and dignity	*“Old windows cannot be closed, the wind howled through them, there is only one toilet, and no way to close the door”*
	*“No conditions to wash, to take a shower. The boiler which is shut down at night, and we have to warm”*
	*“The doctor didn´t asked me permission for vaginal examination”*
	*“I could eat only on the day after birth”*
Emotional support	*“They don’t even come to ask me how the baby is doing, they didn’t even weigh the baby”*
	*“Since I came I did not receive any care for my baby or myself” (mother who had premature home birth, crying while waiting)*
	*“I wanted my partner with me during labour, but it was not allowed”*
Incurred costs	*“For laboratory analyses payment is requested, but there is something you can get for free”*
	*“There are no syringes in the treatment rooms, and if you forgot, you have to find and buy them”*
	*“Why are we paying for maternal care, while the government is emphasizing free care for mothers and babies?”*

## DISCUSSION

The extent of the experience in 25 countries with the QA/QI MN tool and the specific attention paid by the tool to case management provide an unprecedentedly comprehensive and detailed insight into the existing quality gaps in a wide variety of settings, thus contributing to our understanding of the key issues to be addressed by health providers, hospital managers, and decision makers to ensure effective, respectful and dignified health care to all mothers and babies [6,8].

Although quality gaps in MNHC have been documented for specific components of care in many health system contexts, to our knowledge this is the first description of quality gaps along the continuum of care from admission to discharge in a wide array of health system contexts by using a homogeneous approach. A similar approach was used to assess the quality of neonatal and paediatric care in several LMIC countries [17-20], while a study documented gaps in MN care in three countries in Eastern Europe and Central Asia using the first version of the tool [21].

The review allowed to the identification of macro trends in QoC across countries. Serious quality gaps related to all WHO standards [7] were observed. Areas of care which emerged as particularly critical across facilities and countries included: insufficient or incomplete adherence to recommended evidence-based procedures for normal childbirth and at risk maternal and neonatal conditions and complications; excess of inappropriate or unnecessary interventions; insufficient attention to ensure early contact and initiation of breastfeeding; failure to provide respectful care, adequate communication and emotional support to mothers and babies; insufficient infection control; poor use of information generated locally to analyse processes and outcomes. Insufficient provision of life-saving procedures (*too little-too late)* and excessive interventions of unproven efficacy (*too much-too soon)* [22] and lack of respectful, dignified care and of adequate information to patients and their family members were observed in all countries, irrespectively of health systems structure and capacity, thus indicating that there is some convergence in quality gaps even in greatly diverse systems [23]. At the same time, significant differences were observed among facilities belonging to the same health systems, ie, with very similar staffing, infrastructure, equipment and commodities, thus confirming previous observations [17,20,24] and indicating that responsibility for quality gaps cannot be attributed only to system components and that action at system level must be accompanied by action at facility level, where key staff and hospital mangers can make the difference.

The describe tool has four distinctive features, each producing an added value to the QA/QI process.

First, it explores in depth into medical, nursing and organizational procedures from admission to discharge, looking at actual case management while most of currently available quality assessment tools are focused on basic requirements such as staffing, equipment, and protocols [12]. Second, in line with WHO recommendations [6] it includes women’s experience of care by collecting their views and incorporating them in the feedback to hospital managers and staff, who can in this way acquire a new perspective into the QoC they provide [25,26]. Third, it builds local capacity at both facility level, by refreshing and updating health professionals’ knowledge about evidence-based interventions, and at national level, by promoting the creation of a national team of assessors and a sensitization to quality improvement among lead professionals and policy makers. Finally, and most important, the tool is conceived to prompt a move from the first step, ie, analysis, to the second, ie, planning, thus providing the basis for complete quality improvement cycles, a step which is often missing in current maternal and neonatal quality measurements at facility level [12]. It also allows, through the scoring system, a semiquantitative, and easily visualized when the scoring is converted in a color-code, description of the severity of the gap, which is used for prioritizing actions and to provide a baseline to evaluate change over time.

Although none of these features of the tool is innovative per se, yet their combination is not encountered in current QA/QI approaches in MNHC. Among the tools which have been developed and used to assess quality along the continuum of MNH care, most are focusing on the availability of infrastructure, staff, equipment and on their potential capacity to deliver basic or more comprehensive procedures [9,12,27,28]. They are not designed to build awareness among managers and staff at facility level about what can be done better with the available resources, let alone to promote willingness to change, both of which are hardly achieved without participation in the assessment process and findings. UNICEF has recently proposed a comprehensive approach which by many ways is similar to what we have described in that it includes assessment of case management and collects users’ views along the whole continuum of MNHC [29]. This approach, however, is very resource-intensive and does not seem suitable for use at scale, since it requires 7 days of training for the assessment team and 14 days of observation and analysis in each facility, as opposed to 2 days of training and 2 to 3 days for the assessment required by the WHO tool.

The multifaceted potential of the tool needs to be fully understood as well as its limitations, among which the fact that the key component of the assessment, focusing on case-management, remains necessarily based on assessors’ judgment. However, the granularity of the assessment, which is articulated in several observation items for each recommended standard of care, the fact that the assessment is grounded on information derived from multiple sources, such as observed cases, records and interviews, and the cross-referencing between two or more assessors on the same items reduce significantly the subjectivity of the assessment [14,15]. The tool adopts a quantitative scoring scale indicating whether quality gaps represent moderate, serious or very serious threats to the health and dignity of the recipients of care [14,15]. The scoring system is designed to allow prioritization of issues and to appraise and quantify the results of quality cycles within the same facility, not to make comparisons among hospitals within the same country, let alone among countries. It should be stressed that the primary aim is to foster change in each facility rather than to rank facilities. Use for accreditation, however, is among the potentials of the tool, as country-specific minimum score requirements for each aspect of care could be set for certification or for stepwise progression to excellence.

Our review had to face several limitations. We are not able to claim that we included all the existing experiences done with the WHO QA/QI MN tool. In at least one case where we know the assessment was made we could not find the corresponding report. The reports themselves were heterogeneous in the amount of detail they provided so that we had to work on the maximum amount of information that was available in all reports. The scrutiny for identification and reporting of the quality gaps, based on frequency and severity, had to be based on the judgment of two of the Authors, those who could ensure the best knowledge of the process based on direct participation in many of the assessments, although the findings were confirmed by all Authors.

The experience made with the WHO QA/QI MN tool provides compelling evidence about the importance of a competent quality assessment, based on information collected through a variety of sources and performed by a multidisciplinary team of professionals to ensure specific expertise and insights in the process of care. The professional competence provided by the multidisciplinary assessment team provides authoritative technical and scientific support when needed and has the vital role of ensuring a supportive, peer-to-peer attitude throughout the process. This competent professional insight cannot be substituted by checklists, which provide a guide to ensure standard-based scrutiny but do not offer a peer-to-peer exchange focused mainly on case-management. Similarly to internal auditing facilitated by external professionals [30], this insight is key to build awareness about quality gaps, identify causes and actions to remove obstacles to quality of care and build local capacity.

While recognizing that action at all level of the system is needed to ensure quality of care [31], we argue that the experience made with the tools supports the view that QI cycles at facility level should be primarily based on competent assessments performed by multidisciplinary team of professionals and aimed at identifying in great detail the parts of the care pathway which require improvement.

## Additional material

Online Supplementary Document
