# Supplementary material for: Use of a participatory quality assessment and improvement tool for maternal and neonatal hospital care. Part 1: Review of implementation features and observed quality gaps in 25 countries
Source: J Glob Health. 2020 Dec 19;10(2):020432. doi: 10.7189/jogh.10.020432 (PMC7750018; doi:10.7189/jogh.10.020432)
Supplement: Online Supplementary Document [file jogh-10-020432-s001.pdf]

Online Supplementary Document 1. Additional information about the WHO Maternal and Neonatal Quality Assessment and Improvement tool

Structure of the tool

The tool is organized in five main sections: 1) Hospital support services: this section includes an assessment of the physical infrastructure, staff, availability of medicines, equipment and supplies. 2) Case management: this section includes six chapters assessing essential case management practices for the pregnant women, mothers and newborn in the hospital, plus a chapter dedicated to clinical monitoring. Overall, more than 50% of the total items of the tool are related to case management. This section also contains annexes to evaluate the appropriate use of medicines. 3) Policies and organization of services: this section includes four chapters, assessing the existence, quality and use of relevant hospital policies to ensure infection prevention, guidelines development and dissemination, staff training, audit systems, access to hospital and continuity of care, and patient's rights in hospital. 4) Interviews with the staff and interviews with pregnant women and mothers: this section collects information on case management, organizational aspects of care, holistic and respectful care, from the perspective of service users and individual care providers. 5) Feedback of findings and plan for action: the tool includes summary sections to facilitate feedback based on strengths and weaknesses and a framework to guide the meeting with the facility's staff and managers at the end of the assessment in discussing the findings and proposing actions for quality improvement. Each section includes a number of chapters including a number of criteria (see examples in tab 1).

Table 1. Example of tool chapter. Section 2, chapter 3. Cesarean Section.

3.1 Emergency caesarean section can be performed without delay
3.2 Caesarean section is not performed without indication or with inappropriate indication
3.3 Policies to reduce the likelihood of caesarean section are implemented
3.4 Procedures related to caesarean section are in agreement with the international standards
3.5 Surgical technique is appropriate
3.6 Postoperative care of women after caesarean section is appropriate
3.7 Care of women after the first 24 hours is appropriate

Adherence to standards is assessed through a series of items (table 2).

Table 2. Example of items proposed to assess a standard (3.1 Emergency caesarean section can be performed without delay).

1. A protocol is available for emergency CS stating what the staff should do
2. Emergency caesarean section can be performed in less than 30 minutes
3. Theatre is always ready to perform emergency CS (equipment for surgery, electricity...)
4. Operating theatre staff is immediately available for emergency caesarean section
5. Anesthesiologist is immediately available for emergency CS
6. Surgeon is immediately available for emergency CS
7. Laboratory is immediately available and blood test results readily available
8. Blood is readily available if blood transfusion needed
9. 0-negative blood is always present in the facility

Overall, several hundred of care items are included in the tool. All items are supported by references to international guidelines developed by WHO or, in the few instances, by recognized sources of clinical guidelines.

Sources of data

The sources of data for the assessment include: Observation of care practices, equipment and its use in labour and birth area, theatre and recovery area, postnatal area; Documents and records; Written policies, guidelines, and protocols; Clinical logs: Patient records: a minimum number of clinical records, as specified in the tool chapters, is needed to assess how a specific condition is usually managed; interviews with women and with staff. When direct observation of case management it is not possible for some conditions, techniques such as practical exercises and scenarios (e.g. what would you do if…, let's imagine a woman is brought to the hospital with…) may contribute to information on usual provision of care. Observation of services at night can be useful to assess care during childbirth and emergency cases, if not observed during the day. Assessors are instructed to be respectful, seek permission from women and staff, avoid comments, and politely avoid engaging in dialogue/discussion with staff and managers during observation of clinical practice.

Confidential interviews with women are made based on a semi-structured format. A minimum number of interviews is set at each facility based on patient flow (usually no less than ten). The interviews are made with pregnant women, mothers in the post-partum ward and mothers of sick newborns. Health care providers (doctors, midwives and nurses, chief doctors and heads of departments) are also interviewed. The visit is considered over when the information collected is deemed sufficient to allow a reasonable assessment of the quality of care in each main area.

Assessment and scoring

Each assessor attributes a score to each item using the information gathered from different sources. The indicated scoring system is based on 4 possible categories, with the following meaning: *3 = care corresponding to international standards* (no need for improvement or need of minor improvements only) 2 = substandard care but no significant direct hazard to health or violation of human rights (need for some improvement to reach standard care) 1 = *inadequate care with consequent serious health hazards or violation of women's and/or children's rights,* (e.g. omission of evidence based interventions or information with consequent risk to health or violation of human rights of the woman or child (need of substantial improvement to reach standard care) *0 = very poor care with consequent systematic and severe hazards to the health of mothers and/or newborns* (e.g. systematic omission of potentially life-saving interventions or lack of essential safety requisites for key procedures such as CS, blood transfusion, neonatal resuscitation, etc. and need for thorough revision of the specific item or area).
Each assessor also identifies strengths and weaknesses of each chapter belonging to his/her own area of expertise and provides comments on specific issues as well as possible solutions to be discussed in the feedback session. Assessors with the same areas of expertise working together as a sub-team (e.g. midwife and obstetrician observing birth practices) share their views and scores. There may be instances where two or three team members will assess the same department or unit using the section of the tool relevant to their expertise (e.g. the midwife, the neonatologist and the obstetrician will observe a birth from different and complementary perspectives). In most cases, assessors will work in parallel in different departments.
At the end the whole assessors' team meets to discuss findings, agree on the feedback to be given to the facility, focusing on main strengths and weakness, as a basis for draft facility action plan. Besides at facility level, recommendations emerging from the visit are provided also at district, regional, national level, depending on the width and scope of the assessment, to include actions (e.g. drug and commodities procurement, human resources deployment, guidelines and regulations) that

are beyond the capacity of local managers and staff and need to be taken at higher level of the system.

Implementation steps

Table 3 summarizes the steps that need to be taken throughout the quality assessment / quality improvement (QA/QI) process.

Table 3. Main phases of the participatory assessment and implementation steps

Main phases	Steps (to be taken by the leading agency/authority)
Preparatory phase	Preliminary contacts with national and local authorities and partners<br>Adaptation<br>Translation<br>Identification and training of the national assessment team<br>Identification of hospitals to be assessed (if a country-wide assessment is envisaged), sampling if needed.<br>Communication and preliminary agreements with local health authorities and hospital managers
Assessment visit and feedback	Essential data collection about hospital statistics<br>Introductory meeting with H managers and key H staff<br>Task distribution within assessment team and hospital management/key staff<br>Observations & interviews<br>Analysis of findings, discussion among assessors, preparation of feedback<br>Feedback to local staff & managers and draft facility-level plan of action<br>Debriefing feedback to national authorities and development partners, preliminary agreement of priority actions to be taken at national/regional level
Reporting, follow up and reassessment	Formal report to hospital management<br>Formal report to national/local authorities and partners<br>Planning follow-up visits and reassessment<br>Supportive supervision/recall<br>Re-assessment<br>Feedback to local regional and report

Preparatory phase

Key actors and possible partners need to be identified and involved at an early stage. Timelines for the activity and facilities to be assessed are discussed at this stage. A national group of experts,

including all key professionals is identified, including 2 or 3 professionals from each area of expertise (obstetrician gynaecologists, paediatricians/neonatologists, midwives and paediatric/neonatal nurses) to ensure that a pool of national experts is established. At least in the initial phase of a country-wide assessment, it is recommended that an international team of experts works together with a national team, to provide training and coaching in the use of the tool and related guidelines. The international team is usually composed of an obstetrician-gynaecologist, a midwife, a neonatologist and a neonatal nurse, among whom a team leader is appointed. Professionals with capacity to interview women and staff in their own language should also be part of the team. National assessors should not be staff members or have management responsibility for the hospitals that they are going to assess. It is crucial that the members of the national assessment team are recognized professionals, with experience in staff training and supervision, knowledge of the principles of evidence-based health care and WHO recommendations.

The tool can be used in hospitals of different levels, from small district hospitals to tertiary care centres. An adaptation process is envisaged to take into account country-specific health system features (epidemiology, national legislation and programs related to HR, drugs and equipment) and level of the HF. When planning the assessment, the team of national and international assessors identifies the sections of the tool to be used at different levels of care. For example, the chapter on advanced neonatal care is used only where a neonatal intensive care unit is present.

A one-day workshop is necessary to train the assessors in using the tool. A common understanding of the scoring system should be reached using examples. Distribution of the various chapters among the team members needs is agreed upon. Interviewers are also asked to attend the workshop so to fully understand principles and methods of the assessment and the importance of interviewing women and staff.

Preliminary information on the objectives and methods of the assessment, including its supportive, action-oriented approach, and the proposed timetable are communicated to the local health authorities first and then to the management of the facilities to be assessed prior to the visit. The chapters of the first section regarding information about patient flow, mortality and morbidity indicators, availability of commodities and equipment, infrastructure and human resources is sent to the hospital management before the visit with a request for providing the relevant information. Information provided before the visit needs to be checked directly by the assessors so that any inconsistency can be addressed.

A detailed timetable of the assessment visit, including the feedback session is developed by hospital managers and key staff in collaboration with the assessment team leader. The time needed to assess a facility ranges from one and half to three days depending on the hospital size and complexity. The feedback session (usually a two-hour session) is planned at the end of the visit. All staff members able to attend are invited.

Assessment visit and feedback

The visit starts with an introductory briefing to managers and key staff about objectives and methods of the assessment. The presentation emphasizes that the assessment is part of an initiative to identify areas of care that need to be improved, and to support local managers and staff in identifying actions that should be taken at local level and at higher administrative level to improve the QoC. Confidentiality, ethical principles and respect of the rights of patients are emphasized as guiding principles of the assessment. The visit covers all relevant aspects of the services, including wards, pharmacy, laboratory and includes direct assessment of admitted patients, review of medical records, interviews with staff and mothers. It is not necessary to follow the sequence of the chapters. The sequence of the assessment will depend on convenience factors, organizational needs, and on the occurrence of real cases. For example, if there is an emergency or a birth, it is recommended to give priority to the direct observation.

The visit is considered over when the team believes that sufficient information is collected to assess all the items of the tool that are considered applicable to the health service. All sections are then scored by the team according to the scoring criteria and strengths and weaknesses of each subsection (for example care for normal labour or care for sick newborn babies) as a basis for the feedback.

The feedback meeting is held at the end of the assessment and is aimed to involve all staff in discussing the findings and the suggested actions. Assessors present findings in an encouraging way and avoid blaming individuals. Quotes by women and staff who have been interviewed are reported when deemed appropriate to illustrate specific points. Time is allowed time for managers and staff to present their perception of the findings. Actions, which are under the responsibility of local managers and staff, which could be taken to improve the quality of care for those areas and specific items that the assessment indicated as most deficient are identified, prioritizing actions with high potential impact and which appear more feasible based on available resources. A draft plan of action, using a framework provided by the tool, is developed including a timetable and responsibilities of the hospital management and staff members.

Reporting, follow-up and reassessment

After the assessment each facility receives a full report summarizing findings and recommendations. When the assessment is planned as a country-wide and carried out in a sample of facilities, a comprehensive report is delivered to the MoH to indicate areas the need to be strengthened and actions to be prioritized at the wider system level, using the WHO health system framework. Finally, the terms and timelines for reassessment are agreed upon with national authorities and/or developmental partners.

A complete description of the tool structure and assessment methods can be found at http://www.euro.who.int/en/health-topics/Life-stages/maternal-and-newborn-health/publications/2014/hospital-care-for-mothers-and-newborn-babies-quality-assessment-and-improvement-tool.
